# Characterization of anti-drug antibody dynamics using a bivariate mixed hidden-markov model by nonlinear-mixed effects approach

**DOI:** 10.1007/s10928-023-09890-8

**Published:** 2023-11-09

**Authors:** Ari Brekkan, Rocío Lledo-Garcia, Brigitte Lacroix, Siv Jönsson, Mats O. Karlsson, Elodie L. Plan

**Affiliations:** 1 Department of Pharmacy, Uppsala University, Box 580, Uppsala, SE-75123 Sweden; 2 UCB Pharma, Slough, UK; 3 UCB Pharma, Braine l’Alleud, Belgium

**Keywords:** Anti-drug antibody formation, Certolizumab pegol, anti-TNF, hidden-Markov model

## Abstract

Biological therapies may act as immunogenic triggers leading to the formation of anti-drug antibodies (ADAs). Population pharmacokinetic (PK) models can be used to characterize the relationship between ADA and drug disposition but often rely on the ADA bioassay results, which may not be sufficiently sensitive to inform on this characterization.

In this work, a methodology that could help to further elucidate the underlying ADA production and impact on the drug disposition was explored. A mixed hidden-Markov model (MHMM) was developed to characterize the underlying (hidden) formation of ADA against the biologic, using certolizumab pegol (CZP), as a test drug. CZP is a PEGylated Fc free TNF-inhibitor used in the treatment of rheumatoid arthritis and other chronic inflammatory diseases.

The bivariate MHMM used information from plasma drug concentrations and ADA measurements, from six clinical studies (n = 845), that were correlated through a bivariate Gaussian function to infer about two hidden states; production and no-production of ADA influencing PK. Estimation of inter-individual variability was not supported in this case. Parameters associated with the observed part of the model were reasonably well estimated while parameters associated with the hidden part were less precise. Individual state sequences obtained using a Viterbi algorithm suggested that the model was able to determine the start of ADA production for each individual, being a more assay-independent methodology than traditional population PK. The model serves as a basis for identification of covariates influencing the ADA formation, and thus has the potential to identify aspects that minimize its impact on PK and/or efficacy.

**Supplementary Information:**

The online version contains supplementary material available at 10.1007/s10928-023-09890-8.

## Introduction

The administration of biological drugs can invoke an immune response where anti-drug antibodies (ADAs) are formed [1]. Since ADA formed against biological drugs may cause treatment failure, characterization of immunogenic response is important. Formed ADAs may differ with respect to their clinical impact, e.g., not all binding antibodies are neutralizing and some may affect the disposition of biologics via formation of drug-ADA complexes, which may be eliminated differently from the unbound drug [2]. ADA formation also depends on variables specific to the patient/disease, product, or treatment [3, 4]. For instance, concomitant methotrexate (MTX), may reduce the incidence of ADA formation [5]. Route of administration, frequency and dose are examples of treatment specific factors, where ADA formation may be minimized by frequent administrations and/or larger doses of the drug [6].

Biologics and ADA have traditionally been measured using sandwich enzyme-linked immunosorbent assays (ELISA) where high concentrations of the drug can interfere with ADA detection, resulting in false negatives [7]. The characterization of assay drug tolerance is therefore of relevance. Furthermore, some assay methods may not be selective enough for ADA against the drug of interest, resulting in false positives. Although improvements in assay techniques allow for better ADA characterization with higher sensitivity (i.e., drug tolerant, more selective, etc.), the development of such assays may still not add to the interpretation of the ADA in the context of PK and efficacy influence, e.g. ADA are reliably measured but no characterization to classify them as neutralizing available, in such case the occurrence of ADA may add to explain variability in PK but may not have use for clinical interpretation due to the lack of information on whether the ADA is neutralizing or not [8, 9]. Even if improvements in assay techniques result in an increase in the sensitivity of the assay to detect ADA, the priority should be to identify ADA with a clinical impact and to determine thresholds for ADA levels that are clinically relevant. Such determinations can often be susceptible to bias, particularly when the frequency of positive ADA is low and common statistical methods cannot be used to determine a clinically relevant threshold [10].

Methods to characterize ADA dynamics and patient response to ADA are of value given the potential consequences on clinical outcome. Population pharmacokinetic (PK) and pharmacodynamic (PD) analyses are frequently used to characterize the disposition of drugs and several have been published for anti-TNFα therapeutics [11–13]. To account for immunogenicity in such analyses, ADAs are often included as covariates on the clearance (CL) of the therapeutic. However, due to the aforementioned assay aspects, some ADA measurements may be classified as being false positive or false negative. Further, patients are commonly not considered ADA positive until the ADA measurement reaches a threshold level, afterconsequences for drug disposition may already have occurred. Therefore, alternative methods accounting for the masked (or hidden) nature of ADA dynamics and discerning ADA production that influences the PK of the drug may be of interest.

Mixed hidden-Markov models (MHMMs) can be used to determine the relationship between observed and unobservable stochastic processes on a population level [14]. MHMMs allow for the characterization of unobservable processes in patients using one or more observed variables. They have been applied in various areas, including drawing inference about relapse in multiple sclerosis (hidden variable) given observations of brain lesions in patients and determining exposure-response in epilepsy where epileptic activity is the hidden variable [14, 15]. An MHMM using measurements of PK and ADA to inform the immunogenic response (hidden variable), may enable the characterization of the underlying ADA production in patients [16].

The aim of this work was to explore a novel method to characterize ADA dynamics, through the development of a bivariate MHMM accounting for drug PK and ADA measurements to predict the most likely ADA dynamics in an individual, using certolizumab pegol (CZP) data as an example. CZP is a PEGylated Fc-free antibody fragment, which minimizes its potential for complement-dependent cytotoxicity and antibody dependent cell-mediated cytotoxicity [17]. Pegylation combats the relatively short half-life caused by the removal of the Fc region, resulting in a well-tolerated molecule that has a half-life of approximately 14 days [18]. CZP binds to both soluble and membrane-bound TNFα, blocking the TNFα mediated inflammatory cascade and reducing the clinical symptoms of RA and other chronic inflammatory diseases [19]. In the current work, historical ADA binding data and PK was available based on the studies used. New ADA binding and PK assays have been developed for to test the drug in other indications, according to the state of the art and lifecycle management of the product however, for the purpose of the model development, the former historical assays were considered appropriate.

## Methods

### Studies

Data used for model building consisted of five phase II/III trials and one phase I trial where CZP was administered subcutaneously to patients with moderate to severe RA (summarized in Table 1 [18, 31]). PK and ADA were mostly measured simultaneously, see Table 1. Overall, a total of 840 patients with 6898 CZP plasma concentration measurements and 6557 ADA measurements were available for the analysis. The proportion of patients that were reported as being ADA positive (ADA level exceeding the threshold) was 9.8% in the data used for model building. Informed consent was obtained from all study subjects. The studies were conducted in accordance with the applicable regulatory and International Council for Harmonisation–Good Clinical Practice requirements, the ethical principles that have their origin in the Declaration of Helsinki, and the local laws and regulations of the study sites.

**Table 1:** Data used in the analysis

Study^a^	CDP870-004	RA006	CDP870-011	CDP870-014	C87041	PHA001
**Phase**	II	III	III	III	III	I
**n** ^**b**^	239	116	111	124	239	16
**Doses** ^**c**^ **(mg)**	50, 100, 200, 400, 600, 800	400 LD + 200	400	400	200, 400, 400 LD + 200, 200 LD + 100	400
**Dosing frequency**	Q4W	Q2W	Q4W	Q4W	Q2W	Single dose
**PK** **observations/patient (median [range])**	10 [2–10]	7 [4–8]	10 [2–11]	8 [3–11]	7 [2–9]	22 [22–23]
**ADA observations/patient (median [range])**	10 [2–10]	7 [5–8]	10 [2–11]	8 [3–11]	7 [3–9]	Missing
**Planned sampling times (days)**	pre-dose, 7, 14, 28, 35, 42, 56, 63, 70, 84	7, 14, 28, 42, 56, 84, follow-up	7, 14, 28, 56, 84, 112, 140, 147, 154, 168	7, 14, 28, 56, 84, 112, 140, 168	7, 14, 28, 42, 56, 84, 168	0.02, 0.04, 0.08, 0.17, 0.25, 0.33, 0.5, 1, 1.5, 2, 3, 4, 5, 6, 7, 13, 20, 27, 34, 41, 48, 55

ADA, anti-drug antibody; LD, loading dose; PK, pharmacokinetics; Q2W, every second week; Q4W, every fourth week

^a^Have been included in a PKPD meta-analysis previously

^b^Number of patients with CZP concentrations. 2 subjects in CDP870-004 were excluded due to lack of information

^c^In studies **CDP870-004**, **CDP870-011** and **CDP870-014**, no loading dose was administered. Study PHA001 was a single dose study. In studies **RA006** and **C87041**, loading doses were administered at weeks 0, 2, and 4 and followed by the maintenance doses

### Observed variables

Two observed variables were considered; individually weighted PK residuals (${Y}_{{PK}_{RES}}$) and ADA measurements (${Y}_{{ADA}_{MES}}$), to jointly incorporate the information from (i) the PK exposure of CZP as predicted from a model assuming no ADA production and (ii) information from the ADA assay, which is sensitive to the drug. EM-algorithms were used for estimation and parameters were MU-referenced.

To estimate the mode of ${Y}_{{PK}_{RES}}$, the fit from a population PK model to the available CZP concentrations was used. The model was a one-compartment model including covariate effects of weight on CL/F and ethnicity (Japanese) on CL/F and apparent central volume (V/F). ADA was not part of the model. Inter-individual variability (IIV) was present on CL/F and V/F and a proportional residual error model was used. The model was initially fit (parameter estimation) to data from the first dosing occasion, thereafter the obtained individual parameter estimates predicted the remaining dosing occasions. Good model performance, i.e. unbiased residuals, is expected at the first dosing occasion, since patients were drug-naïve and unlikely to have formed ADA impacting the disposition of CZP at this time (last PK observation of first dosing occasion was at 23.6 days [average]). For future dosing occasions and stationary PK characteristics, predictions are also expected to be unbiased resulting in residuals centred around zero. However, in the presence of ADA, the model is expected to over-predict the observed CZP PK at subsequent dosing occasions, due to the absence of a covariate effect of ADA in the model, resulting in negative ${Y}_{{PK}_{RES}}$with a distribution deviating from the expected. Using the ${Y}_{{PK}_{RES}}$ instead of the actual PK observations of CZP as an observed variable was motivated by two factors: ${Y}_{{PK}_{RES}}$have an expected distribution, which can be incorporated in the developed model, and, ${Y}_{{PK}_{RES}}$ give an objective measure of when model performance indicates deviation from stationarity, expected when ADA are formed and not accounted for in the model.

In the studies included, ADAs were measured using a validated ELISA according to standards at the time of the bioanalysis [18, 31], since that time of analysis the ADA assay method has followed the lifecycle management and has been updated in later indications according to the state of art bioassay evolution for ADA detection. Subjects were classified as ADA clinically positive when at least one ADA sample was found to be above a set threshold of 2.4 U/mL. The threshold of 2.4 U/mL was derived based on its apparent clinical relevance where individuals in a phase II study with antibody measurements > 2.4 U/mL had lower CZP systemic exposure than those with measurements < 2.4 U/mL [18, 31]. Following this identification of clinically positive ADA, individuals were further classified as being persistent or transient ADA producers by individual subject profile inspection. The lower limits of quantification (LLOQ) of the CZP and ADA assays were 0.41 µg/mL and 0.6 U/mL, respectively, and all measurements that were below the quantification limit were set to their respective limits.

### Hidden-Markov model development

A two-state MHMM related the observed variables (${Y}_{{PK}_{RES}}$ and ${Y}_{{ADA}_{MES}}$), to the unobserved (hidden) underlying ADA production and the dependency of the observations on previous observations. A subject with a high measurement of ADA will likely have a high measurement at the next time point, thus a first-order Markov element was considered in this work. Production of ADA (*S*_*ADA*_) and no production of ADA (*S*_*NOADA*_) were the two underlying states that were set as a representation of the immune response and the probability of transition between states were estimated. The transition probabilities sum up to 1 and only two elements of the transition probability matrix were estimated: probability of transitioning to *S*_*ADA*_ given that the previous state was *S*_*NOADA (*_*π*_*NOADA−ADA)*_ and probability of transitioning to *S*_*NOADA*_ given that the previous state was *S*_*ADA*_*(π*_*ADA−NOADA*_*)*. he probabilities of remaining in the same state could be derived (*π*_*NOADA−NOADA*_ and *π*_*ADA−ADA*_.) All subjects were assumed to be drug naïve according to the studies criteria and therefore, the stationary distribution vector was not estimated, but rather the model was set up so that everyone started in *S*_*NOADA*_. The general description of an MHMM and its implementation in NONMEM has previously been described in Brekkan et al. [20]. The emission probabilities, which relate the probabilities of the observed variables to the hidden state at that time, were modeled as continuous random variables that could be correlated through a bivariate Gaussian probability density function (PDF):

1 $$\displaylines{P\left( {{Y_{P{K_{RES}}}},{Y_{AD{A_{MES}}}}|S = s} \right) = \frac{1}{{2\pi \sqrt {\sigma _{P{K_{RE{S_s}}}}^2\sigma _{AD{A_{ME{S_s}}}}^2(1 - \rho _s^2)} }}e \cr ^{ - \frac{1}{{2(1 - \rho _s^2)}}\left( {{{\left( {\frac{{{Y_{P{K_{RES}}}} - P{K_{RE{S_{si}}}}}}{{{\sigma _{P{K_{RE{S_s}}}}}}}} \right)}^2} - 2{\rho _s}\left( {\frac{{{Y_{P{K_{RES}}}} - P{K_{RE{S_{si}}}}}}{{{\sigma _{P{K_{RE{S_s}}}}}}}} \right)\left( {\frac{{{Y_{AD{A_{MES}}}} - AD{A_{ME{S_{si}}}}}}{{{\sigma _{AD{A_{ME{S_s}}}}}}}} \right) + {{\left( {\frac{{{Y_{AD{A_{MES}}}} - AD{A_{ME{S_{si}}}}}}{{{\sigma _{AD{A_{MESs}}}}}}} \right)}^2}} \right)} \cr}$$

where${{Y}}_{{{P}{K}}_{{R}{E}{S}}}$ and ${{Y}}_{{{A}{D}{A}}_{{M}{E}{S}}}$ are observed variables of interest, s is the current state (hidden), which can be either *S*_*NOADA*_ or *S*_*ADA*_, ${{P}{K}}_{{R}{E}{S}{s}{i}}$and ${{A}{D}{A}}_{{{M}{E}{S}}_{{s}{i}}}$ are state specific individual model predictions of the variables, ${{\sigma }}_{{{P}{K}}_{{{R}{E}{S}}_{{s}}}}^{2}$ and ${{\sigma }}_{{{A}{D}{A}}_{{{M}{E}{S}}_{{s}}}}^{2}$ are state-specific variances of the variables (residual error) and *ρ*_*s*_ is the correlation between the variables. Two emission probability functions were required, one for each hidden state.

The model was extended to include IIV on several parameters. The individual values for the parameters were modeled assuming a normal distribution, exemplified for *PK*_*RES*_:

2 $${{PK}_{{RES}_{s}}}_{i} ={PK}_{{RES}_{s}}+ {\eta }_{{PK}_{RES}}$$

where *i* denotes an individual,${{PK}_{{RES}_{s}}}_{i}$ is individual estimate of *PK*_*RES*_ in a certain state (*s)*, ${PK}_{{RES}_{s}}$ is the population estimate of the mode of the *PK*_*RES*,_ and ${\eta }_{{PK}_{RES}}$ is a random effect assumed N(0, ${\omega }_{{PK}_{RES}}^{2}$) describing the deviation between the individual and typical values. A logit function was included on all probabilities to prevent individual probabilities exceeding 1 and then back converted to the probabilities using the logistic function, exemplified for *π*_*NOADA−ADA*_:

3 $${\pi }_{{NOADA-ADA}_{i}}=\frac{\text{e}\text{x}\text{p}({\text{L}\text{o}\text{g}\text{i}\text{t}}_{{\pi }_{NOADA-ADA}}+ {\eta }_{{\pi }_{NOADA-ADA}})}{1+ \text{e}\text{x}\text{p}({\text{L}\text{o}\text{g}\text{i}\text{t}}_{{\pi }_{NOADA-ADA}}+ {\eta }_{{\pi }_{NOADA-ADA}})}$$

where ${\pi }_{{NOADA-ADA}_{i}}$ is the individual transition probability of going from *S*_*NOADA*_ to *S*_*ADA*_ and ${\eta }_{{\pi }_{NOADA-ADA}}$ is a random effect assumed N(0, ${\omega }_{{\pi }_{NOADA-ADA}}^{2}$).

**Fig. 1:**
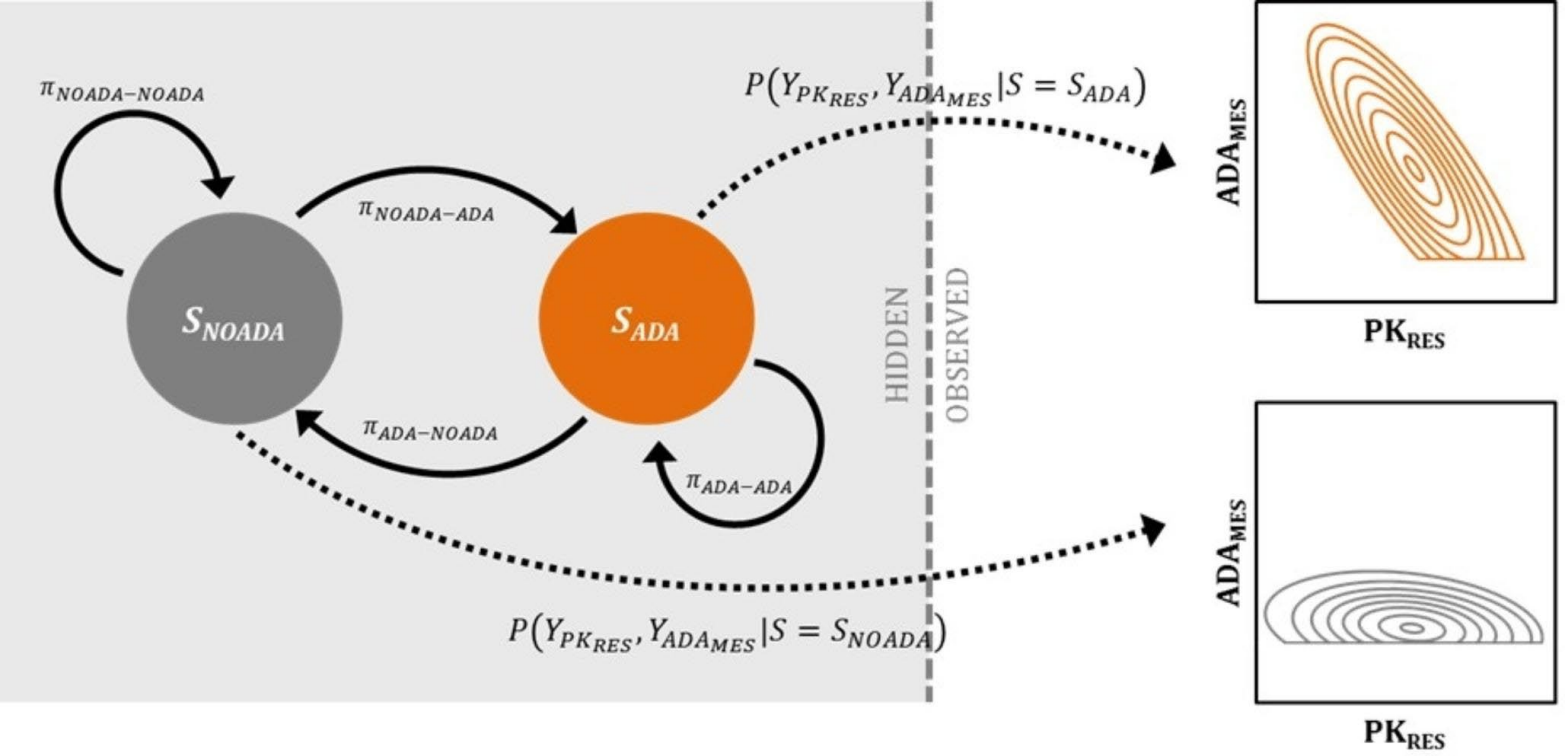
The general model structure of the bivariate hidden-Markov model given pharmacokinetic (PK) observations (${Y}_{{PK}_{RES}}$) and anti-drug antibody measurements (${Y}_{{ADA}_{MES}}$). The two hidden states, *S*_*NOADA*_ (grey) and *S*_*ADA*_ (orange) represent no production of ADA and production of ADA, respectively. The dashed vertical line separates the hidden part of the model from the observable part of the model. Observations were modeled using a bivariate Gaussian function. The probability of transitioning from *S*_*NOADA*_ to *S*_*ADA*_ is *π*_*NOADA−ADA*_ and the probability of transitioning from *S*_*ADA*_ to *S*_*NOADA*_ is *π*_*ADA−NOADA.*_ The probabilities of staying in the respective states are given by *π*_*NOADA−NOADA*_ and *π*_*ADA−ADA*_. The dashed arrows represent the emission probabilities, i.e., the probabilities of the observations given the hidden state (for example $P\left({Y}_{{PK}_{RES}},{Y}_{{ADA}_{MES}}|S={S}_{ADA}\right)$ for the ADA producing state)

The population parameters to be estimated in the model and their expected estimates are presented in Table 2 and the model structure is presented in Fig. 1.

**Table 2:** Parameters in the developed bivariate hidden-Markov model

Parameter	Description	Prior expectation	Rationale for prior expectation
**Observed variable parameters**			
*PK*_*RES*_|*S*_*NOADA*_	Mode of the PK residual in the no production of ADA state	~ 0	When no ADA are produced the PK model should perform well and result in reasonably unbiased residuals.
*PK*_*RES*_ in *S*_*ADA*_	Mode of the PK residual in the production of ADA state	< 0	When ADA are produced the model should over-predict the PK and thus the resulting residuals should be negative.
*ADA*_*MES*_ in *S*_*NOADA*_	Mode of ADA in the no production of ADA state (U/mL)	~ 0.6	When no ADA are produced ADA should be undetectable and thus the estimate should be the LLOQ of the ADA assay.
*ADA*_*MES*_ in *S*_*ADA*_	Mode of ADA in the production of ADA state (U/mL)	≥ 2.4	When ADA that influence PK are produced the ADA assay measurement should be ≥ 2.4 as this is the clinical threshold for ADA positivity.
${\sigma }_{{PK}_{RES}}^{2}$	Variance of PK residual	~ 1	The expectation for well performing weighted residuals is that they should have a standard deviation of 1.
${\sigma }_{A{DA}_{MES}}^{2}$	Variance of ADA (U/mL^2^)	None	The SD of ADA_MES_ is untransformed and thus there is no expectation for this value.
${\rho }_{{S}_{NOADA}}$	Correlations of the observed variables in their respective states.	~ or < 0	When ADA measurements are high then the PK residuals should be highly negative, thus there should be a negative correlation between the two observed variables.
$${\rho }_{{S}_{ADA}}$$			
**Hidden-state parameters**			
*π*_*NOADA−ADA*_	Transition probability from S_NOADA_ to *S*_*ADA*_.	~ 0.05	The total number of clinically positive ADA records was 368 out of 6898 observations (368/6898 = 0.05).
*π*_*ADA−NOADA*_	Transition probability from *S*_*ADA*_ to *S*_*NOADA*_.	~ 0	The data did not indicate the strong presence of transient ADA formation.

First, the bivariate MHMM for *PK*_*RES*_ and *ADA*_*MES*_, was developed. Thereafter, the bivariate model was decoupled into two univariate models for the observed variables to determine the impact of incorporating both observed variables in the model. The individual state sequences resulting from the bivariate model and two univariate models were compared to the clinical ADA classification.

### Parameter estimation and software details

Analysis of the data was performed in NONMEM and consisted of model estimation using maximum likelihood and importance sampling (IMP), followed by a post-hoc step in which the most likely state sequence in every individual was obtained using the Viterbi algorithm. The Viterbi algorithm was obtained as a downloadable subroutine for NONMEM available on the developer’s website [21, 22]. Perl-speaks-NONMEM (PsN) was used for execution and intermediate inspection of runs [23].

The likelihood of the first observation record in the model (assuming just one individual) is calculated as the probability of the observations (both at the same time in the bivariate model) in *S*_*NOADA*_ (Eq. 1). The individual contribution of the states to the entire likelihood is kept and used for the next record. For the second record the likelihood is calculated as follows:

4 $${\varPhi }_{NOADA}=\frac{\left({\varPhi }_{NOADA}\bullet {\pi }_{NOADA-NOADA}+{\varPhi }_{ADA}\bullet {\pi }_{ADA-NOADA}\right)\bullet P({PK}_{RES},{ADA}_{MES}|S={S}_{NOADA})}{Likelihood}$$

5 $${\varPhi }_{ADA}=\frac{\left({\varPhi }_{NOADA}\bullet {\pi }_{NOADA-ADA}+{\varPhi }_{ADA}\bullet {\pi }_{ADA-ADA}\right)\bullet P({PK}_{RES},{ADA}_{MES}|S={S}_{ADA})}{Likelihood}$$

6 $$Likelihood={\varPhi }_{NOADA}+{\varPhi }_{ADA}$$

where *P(PK*_*RES*_, *ADA*_*MES*_*)* is the bivariate Gaussian PDF (in the bivariate model) and ${\pi }_{ADA-NOADA}$ and ${\pi }_{ADA-ADA}$ are the transition probabilities. The individual contribution of the states to the likelihood, ${\varPhi }_{NOADA}$ and ${\varPhi }_{ADA}$, are updated at each record and are defined as the entire likelihood divided by the likelihood of *S*_*NOADA*_ and *S*_*ADA*_, for the two respective states. Therefore, when estimating the model parameters, the likelihood of the data is maximized with respect to the transition probabilities and parameters describing observed variables.

### Model evaluation and model predictions

Model parameter estimates were compared to their prior expectations (Table 2). Furthermore, model performance with regards to termination properties was considered, and models that terminated successfully in NONMEM without errors were considered to perform better than those that did not. Models with successful covariance steps resulting in parameter standard errors (SEs) were also considered to perform better than those that did not provide any parameter SEs. Individual state sequences were obtained from the MHMM and compared to the clinical classification of ADA in a random subset of individuals (n = 12) from study CDP870-004, since this study had the most consistent sampling within the individuals. Simulated (n = 100 simulations) distributions of the observed variables (${Y}_{{PK}_{RES}}$ and ${Y}_{{ADA}_{MES}}$) from the final model were compared to the observed distributions.

In individuals that tested positive for ADA via the assay, the time to positive measurement was calculated and compared with the model predicted first time being in *S*_*ADA*_. The best performing model, with regards to individual state prediction and estimation properties, was transferred into two univariate models and re-estimated. The performance of the resulting univariate models was compared with the bivariate model.

## Results

A total of 6898 CZP plasma concentration measurements and 6557 ADA measurements were available for the analysis. For most records, both ADA and PK were available. However, in study PHA001 no ADA measurements were available as it was a single dose administration study.

Bivariate models with different degree of complexity were developed:

- Model (1) Modes of *PK*_*RES*_ and *ADA*_*MES*_ estimated. IIV estimated on modes and transition probabilities.
- Model (2) Modes of *PK*_*RES*_ estimated. *ADA*_*MES*_ modes fixed to clinically relevant values of 0.6 and 2.4 U/mL in *S*_*NOADA*_ and *S*_*ADA*_, respectively. IIV estimated on modes and transition probabilities.
- Model (3) Modes of both *PK*_*RES*_ and *ADA*_*MES*_ estimated. IIV estimated on transition probabilities.
- Model (4) Modes of *PK*_*RES*_ estimated. *ADA*_*MES*_ modes fixed to 0.6 and 2.4 U/mL in *S*_*NOADA*_ and *S*_*ADA*_, respectively. IIV estimated on transition probabilities.
- Model (5) Modes of *PK*_*RES*_ and *ADA*_*MES*_ estimated. No IIV estimated.
- Model (6) Modes of *PK*_*RES*_ estimated. *ADA*_*MES*_ modes fixed to 0.6 and 2.4 U/mL in *S*_*NOADA*_ and *S*_*ADA*_, respectively. No IIV estimated.

**Fig. 2:**
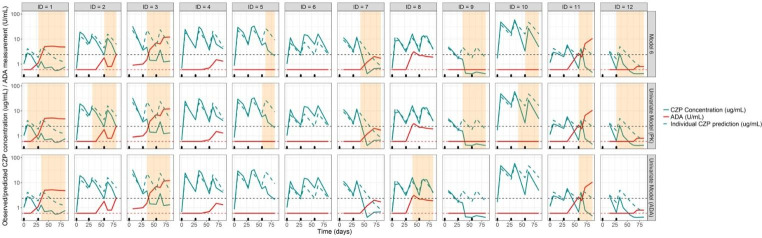
Individual state predictions in twelve individuals resulting from model 6 and the same model only considering univariate observed variables (either PK residuals [middle panels] or ADA measurements [lower panel]). CZP concentration measurements (green), individual CZP PK model predictions (green dashed line) and ADA measurements (red) are presented for 12 selected individuals. Each individual is represented in one panel. The black horizontal dashed line indicates the threshold for clinical positivity (2.4 IU/mL) for the ADA measurement and the red dashed line is the lower limit of quantification for ADA (0.6 IU/mL). The black tick marks are dosing events. The grey shaded area shows when the model predicted a state associated with the production of ADA (S_ADA_).

**Fig. 3:**
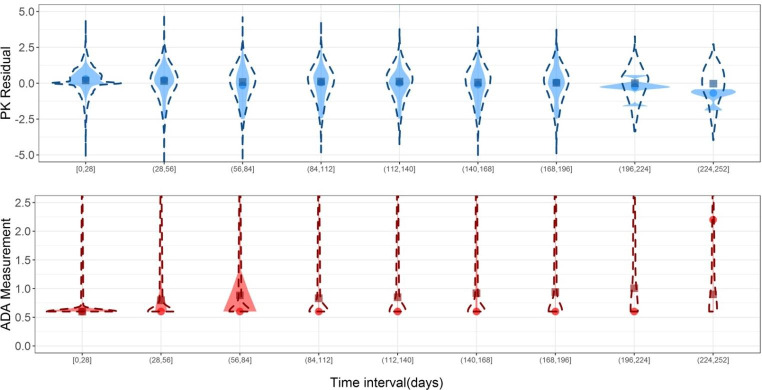
Violin plots of the observed variables (shaded areas and circles [indicate medians]) and simulations from the final model (model 6) (dashed lines and squares [indicate medians]) versus time intervals. Simulated ADA measurements below 0.6 were set to 0.6 as LOQ.

Model 6 was the best performing model as indicated by individual state sequence predictions (Fig. 2) and parameter estimation. Parameter estimates of the final model are presented in Table 3. Simulations of the dependent variables from model 6 are presented in Fig. 3 and in Supplemental 4 and show that ${Y}_{{PK}_{RES}}$were well captured while ${Y}_{{ADA}_{MES}}$ were not as well captured.

**Table 3:** Parameter estimates of the final bivariate model. FIX indicated parameters which were not estimated

Parameter	Prior expectation		Estimate (RSE%)
**Observed variable parameters**			
*PK*_*RES*_ in *S*_*NOADA*_	~ 0		0.3 (6)
*PK*_*RES*_ in *S*_*ADA*_	< 0		-1.6 (3)
*ADA*_*MES*_ in *S*_*NOADA*_	~ 0.6		0.6 FIX
*ADA*_*MES*_ in *S*_*ADA*_	≥ 2.4		2.4 FIX
${\sigma }_{{PK}_{RES}}^{2}$	1		0.8 (13)
${\sigma }_{ADA}^{2}$	None		1.6 (19)
${\rho }_{{S}_{NOADA}}$	< 0 or ~ 0		-0.1 (32)
${\rho }_{{S}_{ADA}}$	< 0		-0.07 (18)
**Hidden variable parameters**			
*π*_*NOADA−ADA*_	~ 0.05		0.03 (12)
*π*_*ADA−NOADA*_		~ 0	0.003 (112)

All models performed well with regards to the observed variable parameter estimates, which were consistent with the expectations (Supplemental 2). However, models 1 to 3 resulted in estimates of π_NOADA−ADA_ at the lower boundary (i.e., ~ 0). Models 4 to 6 had more realistic estimates with π_NOADA−ADA_ of 0.04, 0.02 and 0.03 for these models. In general, for models including IIV, estimation of imprecision in parameters was unsuccessful. The estimation of ADA_MES_ (models 1, 3 and 5) was associated with poor individual state sequence predictions as these models did not predict a transition to S_ADA_ in individuals 5, 7, 9 and 12 despite the indication of ADA formation based on the PK data (Supplemental 3, Figures S3-1, S3-3, S3-5). Models 2 and 5 also performed poorly for these individuals, as expected given the lower boundary estimate of π_NOADA−ADA_ (model 2) while less so for model 5 which resulted in relatively well estimated parameters (apart from ${\rho }_{{S}_{ADA}}$, RSE 110%). Models 4 and 6 performed the best with regards to the individual state sequence predictions, for model 6 predictions are shown in Fig. 2. The RSE of π_ADA−NOADA_ was high (111.5%) in model 6, which is plausible given the low parameter estimate (0.003), indicates that transient ADA formation in the current dataset is unlikely and once an individual is in S_ADA_ they are unlikely to transition to S_NOADA_. Fixing the modes of ADA in both S_NOADA_ and S_ADA_ to values associated with the clinical classification of ADA resulted in improved individual state sequence predictions as indicated by the difference between the predictions from model 5 (Figure S2-4), where the parameters were estimated, and model 6 (Fig. 2), where they were fixed. Simulations from model 6 (Figure S4-1 and S4-2) indicated that ${Y}_{{PK}_{RES}}$ were well captured while ${Y}_{{ADA}_{MES}}$ were not as well captured since the simulated percentiles did not correspond with the same observed percentiles. This is likely due to the sparse nature of ADA observations and because the range of ADA measurements was large.

The mean time to first clinically positive ADA measurement (ADA measurement > 2.4 U/mL) was 81.0 days. The developed models 1 to 6 predicted the mean first time points of a transition from *S*_*NOADA*_ to *S*_*ADA*_ were 85, 78, 83, 64, 84 and 63 days, respectively.

Reducing model 6 to two univariate models showed that the parameter estimates in the two univariate models were, in general, similar to those obtained in the bivariate model, apart from the hidden variable parameters for the univariate *ADA*_*MES*_ model (Table 4). With respect to individual state sequence predictions Model 6 and the univariate *PK*_*RES*_ model performed similarly. However, the univariate *ADA*_*MES*_ model, did not predict a transition to *S*_*ADA*_ for six individuals although their PK indicated presence of ADA, and predicted an individual which may have a false-positive ADA record (occurring at ~ 28 h) as an ADA producer (Fig. 2).

**Table 4:** Parameter estimates of the bivariate model and the two univariate models handling only PK residuals or ADA measurements. Relative standard errors (RSE%) are reported when they were obtained using covariance step in NONMEM with default settings

Estimated parameter	Model 6 parameter estimates (RSE%)	Univariate (PK_RES_) model based on model 6 parameter estimates (RSE%)	Univariate (ADA_MES_) model based on model 6 parameter estimates (RSE%)
**Observed variable parameters**			
*PK*_*RES*_ in *S*_*NOADA*_	0.3 (6)	0.4 (4)	NA
*PK*_*RES*_ in *S*_*ADA*_	-1.6 (3)	-1.4 (4)	NA
ADA in *S*_*NOADA*_	0.6 FIX	NA	0.6 FIX
ADA in *S*_*ADA*_	2.4 FIX	NA	2.4 FIX
${\sigma }_{{PK}_{RES}}^{2}$	0.8 (13)	0.8 (14)	NA
${\sigma }_{ADA}^{2}$	1.6 (19)	NA	1.6 (19)
${\rho }_{{S}_{NOADA}}$	-0.1 (32)	NA	NA
${\rho }_{{S}_{ADA}}$	-0.07 (18)	NA	NA
**Hidden variable parameters**			
*π*_*NOADA−ADA*_	0.03 (12)	0.05 (8)	0.2 (9)
*π*_*ADA−NOADA*_	0.003 (112)	0.005 (146)	1E-5 (0)

## Discussion

A common manifestation of immunogenicity is the formation of ADAs that bind to the therapeutic agent and may cause changes in the disposition of the drug and/or prevent binding to the target reducing the drug effect. ADA against biological therapeutics are routinely characterized and reported as they can be associated with treatment failure [24–26]. The characterization and interpretation of ADA effects on disposition and efficacy of biologics can be limited by the characteristics of the bioassay used to quantify ADAs. For instance, in the presence of excess drug, the commonly used ELISA sandwich assay, may not be drug tolerant resulting in inadequate detection of ADA in the presence of high concentrations of drug [32]. The presence of ADA needs to be put in the context of its effects on clinical efficacy, safety and disposition of the drug [10]. A model, such as the one presented in this work, allows us to gain additional insight into the assessment and characterization of ADA by using all sources of information. Similarly, the developed methodology was applied to ADA against CZP that does not distinguish between antibodies isotypes. In the case where specific isotypes against PEG are measured, the model could be extended to also account for different types of anti-drug antibodies. A caveat in the current analysis is that any discrepancies in PK resulting in changes in the PK residuals are assumed to be attributed to ADA formation; if the model was miss-specified, remaining unexplained PK influences would still be attributed to ADA formation.The parameters of the model could be classified as “observed variable” and “hidden variable” parameters. Two modes were estimated for each of the observed variables, one for each state together, with a single residual term shared across states. A single residual term (per variable) was used in order to reduce model flexibility and assumes that the distribution of the observations were not different depending on which state the observations arose from. If two residual terms would have been estimated for the *ADA*_*MES*_, the residual term related to *S*_*NOADA*_ would have been estimated to ~ 0, as most observations would be equal to the LOQ and the other would be some large value, as measurements of ADA are highly variable. Thus, numerical issues arise as the likelihood would approach infinity due to the division of an increasingly small number in Eq. 3. This may be a consequence of the assumption of observations being equal to LLOQ, instead of recognizing that they are not measurable with the analysis instrument used. In this work, we assumed that the observed variables were normally distributed. The Gaussian distribution was deemed to be appropriate, since for well performing PK models the distribution of the residuals (which we used as the observed variable in the MHMM) are assumed to be normal. For the ADA observations, there is no appropriate distribution as a vast majority of them were below the LOQ and thus set to the LOQ value of 0.6 U/mL. Simulations of the observed variables from the model demonstrated appropriateness of the model for the PK residuals, but simulations of ADA measurements did not perform as well, and underprediction of the number of values associated with high ADA measurements was seen. Since the two residual terms were shared for *ADA*_*MES*,_ the very low variance of ADA in *S*_*NOADA*_ may have contributed to the relatively low estimate of ${\sigma }_{ADA}^{2}$, likely responsible for this behaviour. To correlate *ADA*_*MES*_ with *PK*_*RES*,_ a bivariate normal distribution was assumed. Further, it was assumed that all individuals start in *S*_*NOADA*_, since the individuals included in the trial data were drug naïve.

Fixed effects parameter estimates for the observed variables in the tested bivariate models were as expected in most cases. In several of the models, the modes of *ADA*_*MES*_ were fixed to clinically relevant values. A measurement that is LOQ is most likely associated with no ADA production, while a value greater than 2.4 U/mL is likely to be associated with a true ADA measurement. In our work, estimation of the modes of *ADA*_*MES*_ in the respective states were associated with poorer individual state sequence predictions than when fixing those parameter values. Consequently, by fixing the parameters in the developed models, a weighting of the observations is made, as observations > 2.4 U/mL are more likely to have come from *S*_*ADA*_than *S*_*NOADA*_. For instance, when ${\mu }_{ADA}$ in *S*_*ADA*_ was fixed to 1000 U/mL in a model considering only ADA measurements (univariate model), while estimating the rest of the model parameters, the model did not result in any transitions to *S*_*ADA*_ and thus any transitions in the bivariate model would be driven solely by *PK*_*RES*_ (results not shown). Transition probabilities in the model were generallylow, expected given the few observations associated with ADA formation. Furthermore, the estimate of *π*_*ADA−NOADA*_ was in general much lower than *π*_*NOADA−ADA*_ suggesting a lack of transient ADA formation going from *S*_*NOADA*_ to *S*_*ADA*_ and back again for an individual.

The estimation of IIV was associated with both issues related to parameter estimation and worse individual state sequence predictions. Although inclusion of IIV on the transition probabilities makes sense, this was poorly estimated given the data, with only very few transitions in the total data, and very few individuals having transient ADA formation. This rendered the estimation of additional parameters (such as IIV) not possible. IIV on the transition probabilities would allow covariates influencing the ADA status to be identified. This is of interest, to reduce the probability of transitioning to *S*_*ADA*_, which is in turn associated with a more beneficial treatment outcome. For instance, MTX administration can reduce the incidence of ADA formation, and this may be tested in the MHMM. However, this would require a larger number of transitions within an individual to be viable. The current model was estimated to a large data set. Further research is required to determine the data requirements for state-sequence predictions in smaller data sets generated in clinical practice.

When ADA are included in population PK analyses, they are often included as a dichotomous or continuous covariate on drug CL [28–30]. These models often do not consider a time-course of ADA positivity since a subject who is ADA positive at a certain timepoint is assumed to remain positive throughout the analysis. It may be possible to use the information obtained from the present model to infer the probabilities of being in an ADA producing state and using those resulting probabilities as a covariate in the PK model. The time-course of ADA can be more readily incorporated since ADA are not the only component driving the PK response.

In the best performing model (model 6, Table 3; Fig. 2), the individual state sequence predictions obtained for a subset of individuals was able to identify potential false positive and negatives, as well as to assign ADA positive status influencing PK before the ADA was detected, and therefore before the onset of ADA effects based on the combined information coming from the *PK*_*RES*_ and *ADA*_*MES*_. This highlights that the present model, by using all the information available, may fill the caveats related to the ADA bioassay that make it difficult to obtain a true characterization of immunogenicity in some instances. The modelling results may suggest that ADA are formed earlier than the assay can detect them. In individuals that tested positive for ADA, the mean time to measuring ADA was 81 days while the mean model predicted time to transitioning to the ADA producing state was 63 days. These results depend on the number of observations available, where more observations would result in more individual state sequence predictions. This methodology may be able to identify false-negative ADA measurements, and thus better relate ADA to potential clinical impact as well as limit the otherwise noise risked when classical population PK methods without characterisation of the ADA is performed; whereas identifying false-positive measurements may not be as relevant if PK is unaffected. The model performed well to correct for some false-negative ADA measurements and was able to suggest false-positives as well. In the current work, ADA measurements alone could not result in the observed state-sequence predictions. The model requires a second observed variable containing information about the impact of ADA on PK, which could then provide further information on potential clinical relevance (for example, if efficacy was impacted). Without the PK residual as a second observed variable in the current model, drawing any conclusions regarding clinical relevance would be difficult. Further work may be needed to elucidate what types of observations are most informative for clinical relevance purposes in the context of ADA assay results interpretation.

## Conclusion

The immunogenic response to proteins is heterogeneous and depends on different factors inherent to the drug, patient, disease and others such as dose regimen and route of administration. Characterization of ADA and its impact on PK, efficacy and safety is key in drug development of new entities. PKPD models often focus on characterising the impact of ADA on PK and hence clinical consequences. However, they do not focus on what triggers the ADA response.

We developed a model able to characterise the hidden ADA production and state transitions using all the sources of information available (both PK and ADA). To our knowledge, this is the first time that this methodology has been applied to characterize ADA, and this work has served as a basis for other posterior examples that have recently been published [33, 34]. The results presented in this work should serve as an example for the use of a MHMM in the analysis of immunogenicity that makes use of all available PK and ADA data, and is able to weigh in the information being more assay independent than traditional population PK analysis exploration of ADA. Although not explored here, future uses of the model may include the identification of covariates influencing the transition probabilities in the model that would help in identifying trial design and treatment related aspects that would minimize the ADA formation and thus minimize its impact on PK and/or efficacy. The methodology can be extended to other molecular entities and be used to inform on covariates that trigger immunogenicity response. Further it can be linked back to the clinical implication on safety and efficacy, thereby inform on interventions that would be required to reduce immunogenicity response to a drug.

### Electronic supplementary material

Below is the link to the electronic supplementary material.

Supplementary Material 1
