# Supplementary material for: Characterization of anti-drug antibody dynamics using a bivariate mixed hidden-markov model by nonlinear-mixed effects approach
Source: J Pharmacokinet Pharmacodyn. 2023 Nov 9;51(1):65–75. doi: 10.1007/s10928-023-09890-8 (PMC10884144; doi:10.1007/s10928-023-09890-8)
Supplement: Supplementary file 1 — Supplementary Material 1 [file 10928_2023_9890_MOESM1_ESM.docx]

**Title:** Characterization of Anti-Drug Antibody Dynamics Using a Bivariate Mixed Hidden-Markov Model by Nonlinear-Mixed effects approach

**Running title:** Characterization of ADA dynamics using a bivariate Mixed Hidden-Markov Model (MHMM)

**Journal:** *Journal of Pharmacokinetics and Pharmacodynamics*

**Authors:** Ari Brekkan^1^, Rocío Lledo-Garcia^2^, Brigitte Lacroix^3^, Siv Jönsson^1^, Mats O. Karlsson^1^, Elodie L. Plan^1^

**^1^**Department of Pharmacy, Uppsala University, Box 580, SE-75123, Uppsala, Sweden

**^2^**UCB Pharma, Slough, United Kingdom

**^3^**UCB Pharma, Braine l’Alleud, Belgium

**SUPPLEMENTARY INFORMATION**

**Supplementary information 1**

*Table -S1: Parameter estimates of bivariate models. Relative standard errors (RSE%) are reported when they were obtained using covariance step in NONMEM with default settings.*

| Estimated parameter | Prior expectation | | Model 1 parameter estimates (RSE%) | Model 2 parameter estimates (RSE%) | Model 3 parameter estimates (RSE%) | Model 4 parameter estimates (RSE%) | Model 5 parameter estimates (RSE%) | Model 6 parameter estimates (RSE%) |  |
| --- | --- | --- | --- | --- | --- | --- | --- | --- | --- |
| **Observed variable parameters** | | | | | | | | | |
| *PK_RES_* in *S_NOADA_* | ~ 0 | | 0.2 | 0.4 | 0.2 | 0.3 | 0.2 (10.1) | 0.3 (5.5) |  |
| *PK_RES_* in *S_ADA_* | < 0 | | -0.5 | -1.5 | -1.9 | -1.5 | -1.9 (3.3) | -1.6 (2.6) |  |
| *ADA* in *S_NOADA_* | ~0.6 | | 0.7 | 0.6 FIX | 0.7 | 0.6 FIX | 0.7 (1.7) | 0.6 FIX |  |
| *ADA* in *S_ADA_* | ≥2.4 | | 5.8 | 2.4 FIX | 8.1 | 2.4 FIX | 8.0 (7.3) | 2.4 FIX |  |
| $\sigma_{{PK}_{RES}}^{2}$ | 1 | | 0.6 | 0.6 | 1.2 | 0.8 | 1.2 (8.1) | 0.8 (13.4) |  |
| $\sigma_{ADA}^{2}$ | None | | 0.3 | 0.3 | 0.5 | 1.9 | 0.5 (12.1) | 1.6 (18.7) |  |
| $\rho_{S_{NOADA}}$ | < 0 or ~ 0 | | -0.4 | -0.3 | -0.5 | -0.1 | -0.5 (6.4) | -0.1 (32.1) |  |
| $\rho_{S_{ADA}}$ | < 0 | | -0.02 | -0.03 | -0.008 | -0.08 | -0.008 (109.6) | -0.07 (18.2) |  |
| **Hidden variable parameters** | | | | | | | | | |
| *π_NOADA-ADA_* | | ~ 0.05 | 1E-5* | 1E-5* | 1E-5* | 0.04 | 0.02 (11.6) | 0.03 (12.3) |  |
| *π_ADA-NOADA_* | | ~0 | 1E-5* | 0.2 | 0.2 | 0.002 | 0.2 (21.0) | 0.003 (111.5) |  |
| **Inter-individual variability (reported as variance)** | | | | | | | | | |
| $\omega$ *PK_RES_* in *S_NOADA_* | | None | 0.2 | 1.8 | - | - | - | - |  |
| $\omega$ *PK_RES_* in *S_ADA_* | | None | 12 | 0.5 | - | - | - | - |  |
| $\omega$ *ADA_MES_* in *S_NOADA_* | | None | 0.05 | 0.03 | - | - | - | - |  |
| $\omega$*ADA_MES_* in *S_ADA_* | | None | 51 | 36 | - | - | - | - |  |
| $\omega$ *π_NOADA-ADA_* | | None | 0.4 | 0.4 | 0.3 | 0.07 | - | - |  |
| $\omega$ *π_ADA-NOADA_* | | None | 68 | 0.9 | 0.1 | 01 | - | - |  |

**Supplementary information 2**

**Model 1 individual state sequence predictions**

**
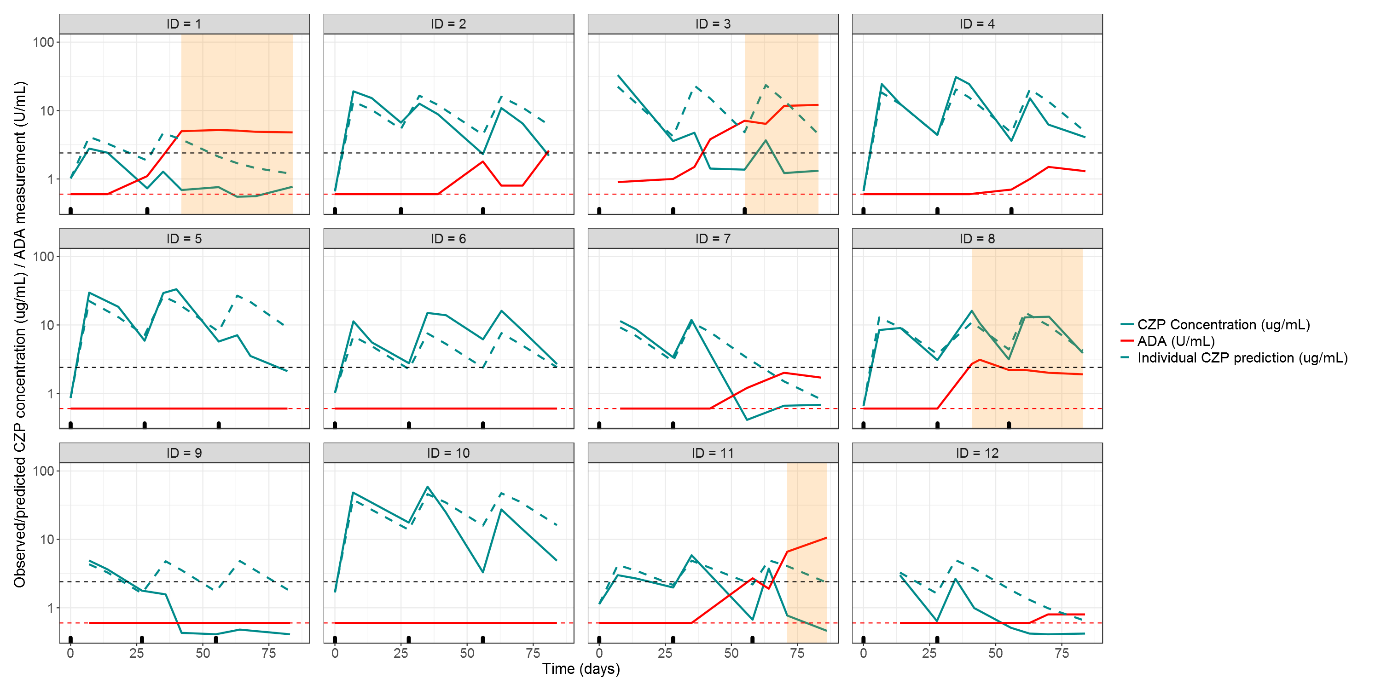
**

***Figure S2-1***: *Individual state predictions in ten individuals resulting from model 1. CZP concentration measurements (green), individual CZP PK model predictions (green dashed line) and ADA measurements (red) are presented for 12 selected individuals. Each individual is represented in one panel. The black horizontal dashed line indicates the threshold for clinical positivity (2.4 IU/mL) for the ADA measurement and the red dashed line is the lower limit of quantification for ADA (0.6 IU/mL). The black tick marks are dosing events. The orange shaded area shows when the model predicted a state associated with the production of ADA (S_ADA_).*

**Model 2 individual state sequence predictions**

**
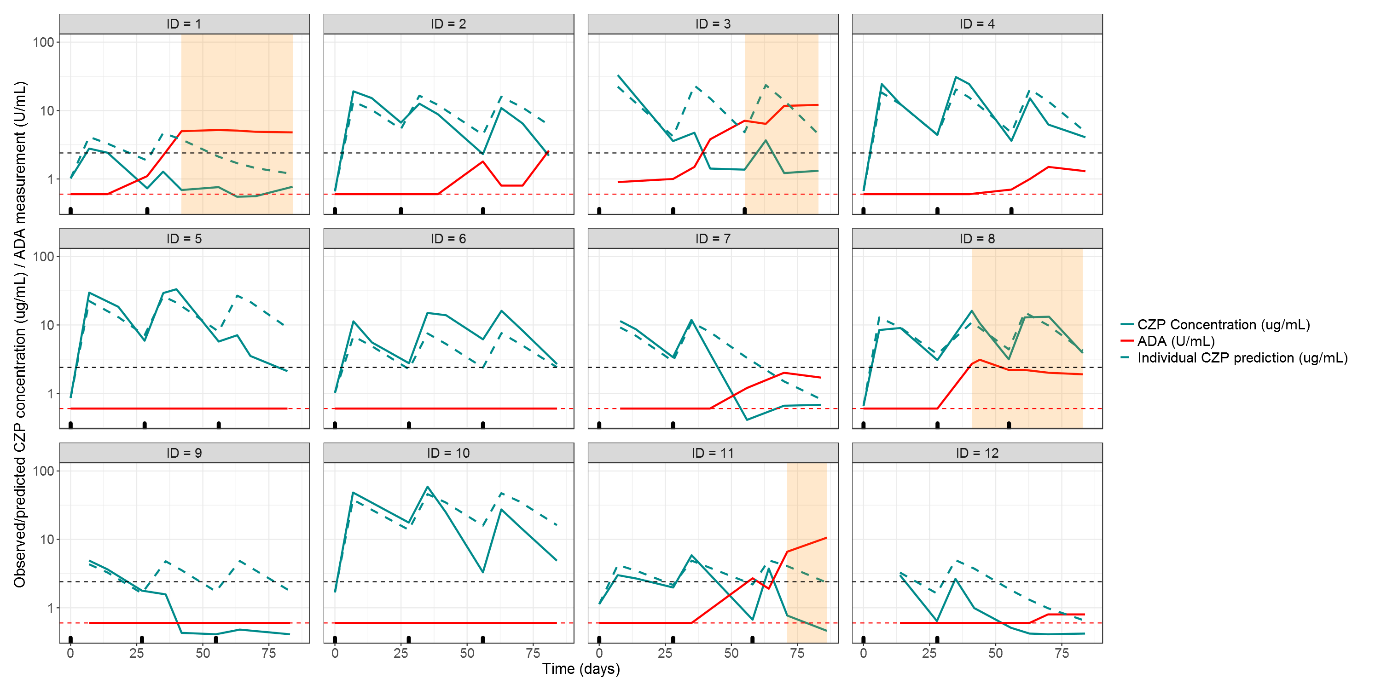
**

***Figure S2-2:*** *Individual state predictions in ten individuals resulting from model 2. CZP concentration measurements (green), individual CZP PK model predictions (green dashed line) and ADA measurements (red) are presented for 12 selected individuals. Each individual is represented in one panel. The black horizontal dashed line indicates the threshold for clinical positivity (2.4 IU/mL) for the ADA measurement and the red dashed line is the lower limit of quantification for ADA (0.6 IU/mL). The black tick marks are dosing events. The orange shaded area shows when the model predicted a state associated with the production of ADA (S_ADA_).*

**Model 3 individual state sequence predictions**

**
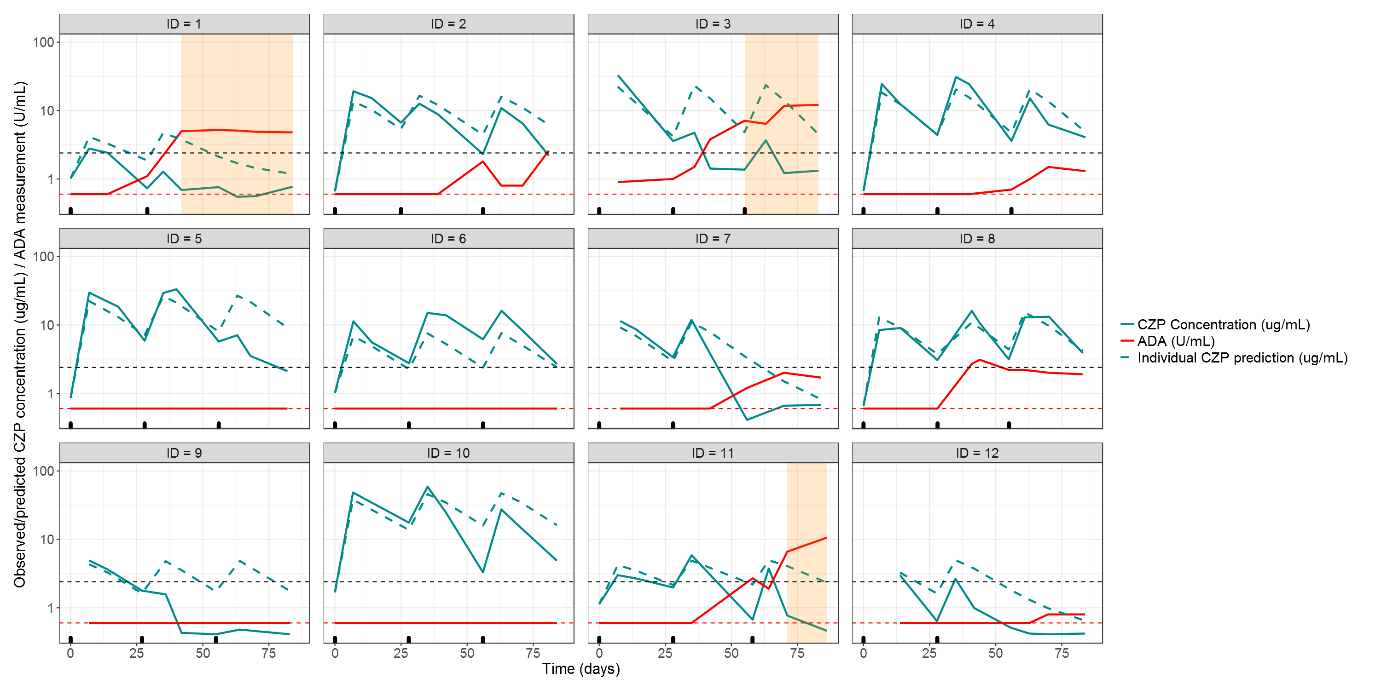
**

***Figure S2-3:*** *Individual state predictions in ten individuals resulting from model 3. CZP concentration measurements (green), individual CZP PK model predictions (green dashed line) and ADA measurements (red) are presented for 12 selected individuals. Each individual is represented in one panel. The black horizontal dashed line indicates the threshold for clinical positivity (2.4 IU/mL) for the ADA measurement and the red dashed line is the lower limit of quantification for ADA (0.6 IU/mL). The black tick marks are dosing events. The orange shaded area shows when the model predicted a state associated with the production of ADA (S_ADA_).*

Model 4 individual state sequence predictions were identical to those obtained from model 6 for the presented individuals (Figure S2-5).

**Model 5 individual state sequence predictions**

**
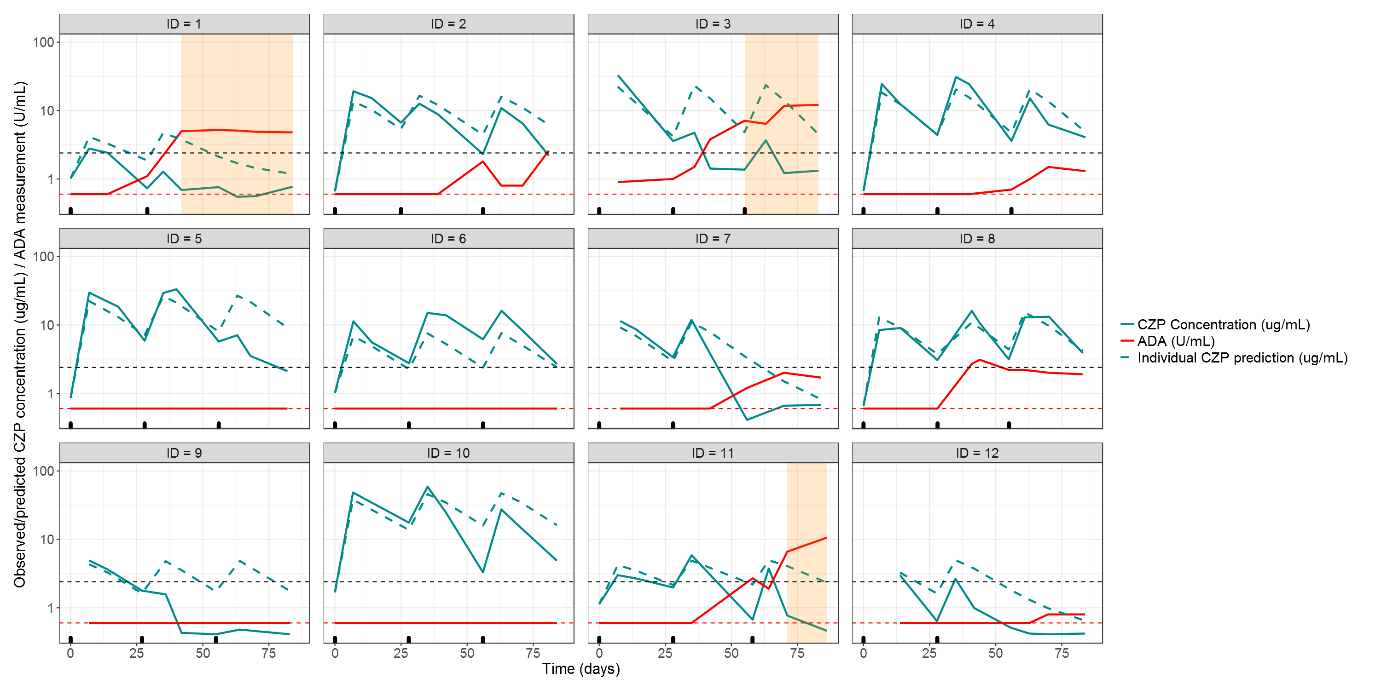
**

***Figure S2-4:*** *Individual state predictions in ten individuals resulting from model 5. CZP concentration measurements (green), individual CZP PK model predictions (green dashed line) and ADA measurements (red) are presented for 12 selected individuals. Each individual is represented in one panel. The black horizontal dashed line indicates the threshold for clinical positivity (2.4 IU/mL) for the ADA measurement and the red dashed line is the lower limit of quantification for ADA (0.6 IU/mL). The black tick marks are dosing events. The orange shaded area shows when the model predicted a state associated with the production of ADA (S_ADA_).*

**Model 6 individual state sequence predictions**


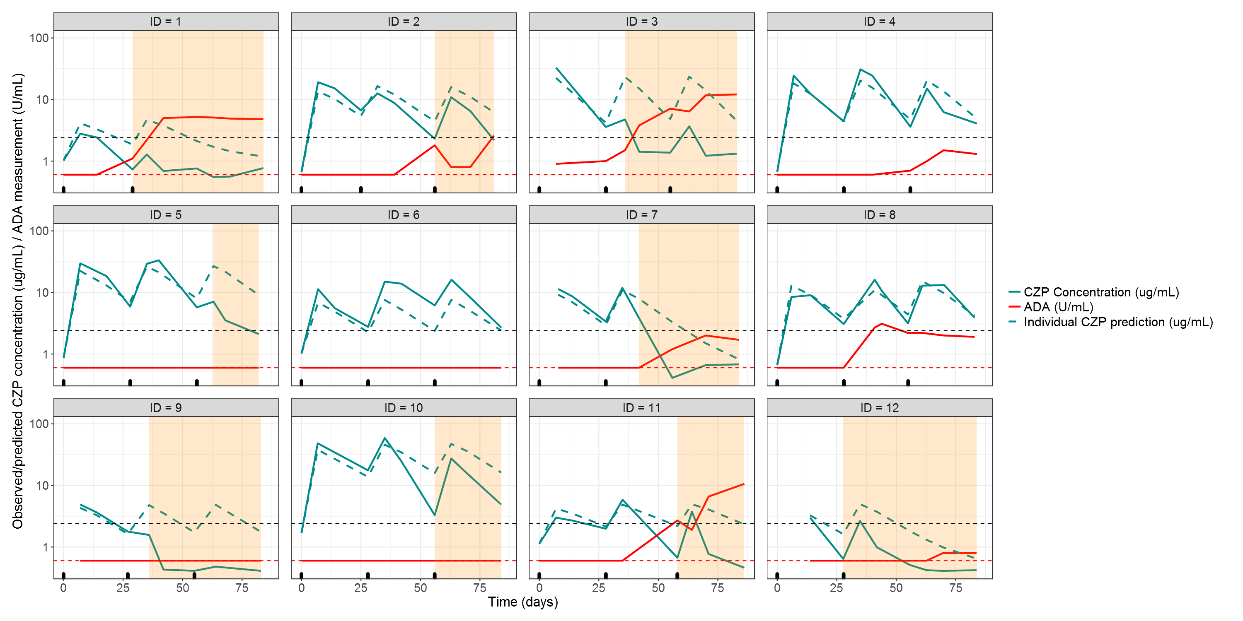


***Figure S2-5:*** *Individual state predictions resulting from the final (model 6) bivariate hidden-Markov model. CZP concentration measurements (green), individual CZP PK model predictions based on EBEs from the first occasion fit (green dashed line) and ADA measurements (red) are presented for 12 random individuals. Each individual is represented in one panel. The black horizontal dashed line indicates the threshold for clinical positivity (2.4 IU/mL) for the ADA measurement and the red dashed line is the lower limit of quantification for ADA (0.6 IU/mL). The black tick marks are dosing events. The orange shaded area shows when the model predicted a state associated with the production of ADA (SADA). For instance, for ID=6, the model did not predict a transition to the ADA producing state and ID=7 was identified as being a potential false negative by the model.*

**Supplementary information 3**

**Model 1 individual state sequence predictions (with *PK_RES_*)**

**
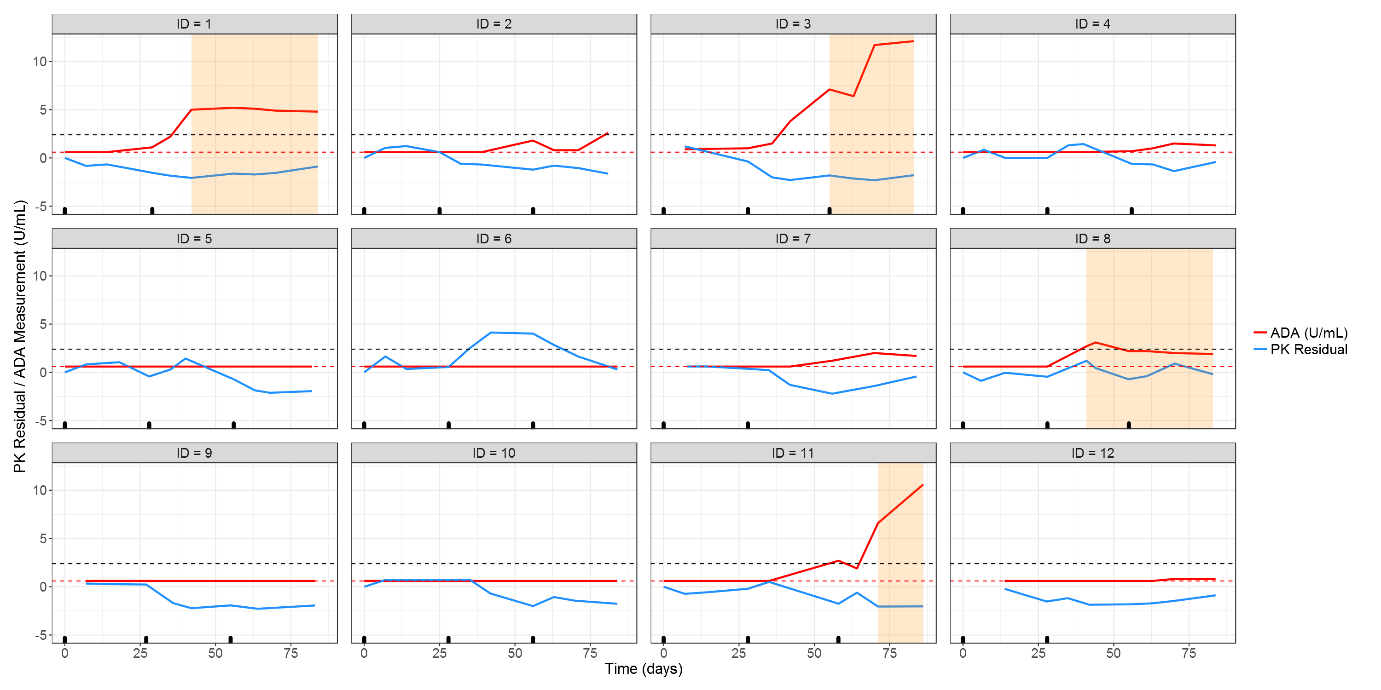
**

***Figure S3-1***: *Individual state predictions in ten individuals resulting from model 1. Individual weighted residuals (PK residual, blue) and ADA measurements (red) are presented for 12 selected individuals. Each individual is represented in each panel. The black dashed line indicates the threshold for clinical positivity (2.4 IU/mL) for the ADA measurement and the red dashed line is the lower limit of quantification for ADA (0.6 IU/mL). The black tick marks are dosing events. The orange shaded area indicates time points which the models predicted a state associated with the production of ADA (S_ADA_).*

**Model 2 individual state sequence predictions (with *PK_RES_*)**

**
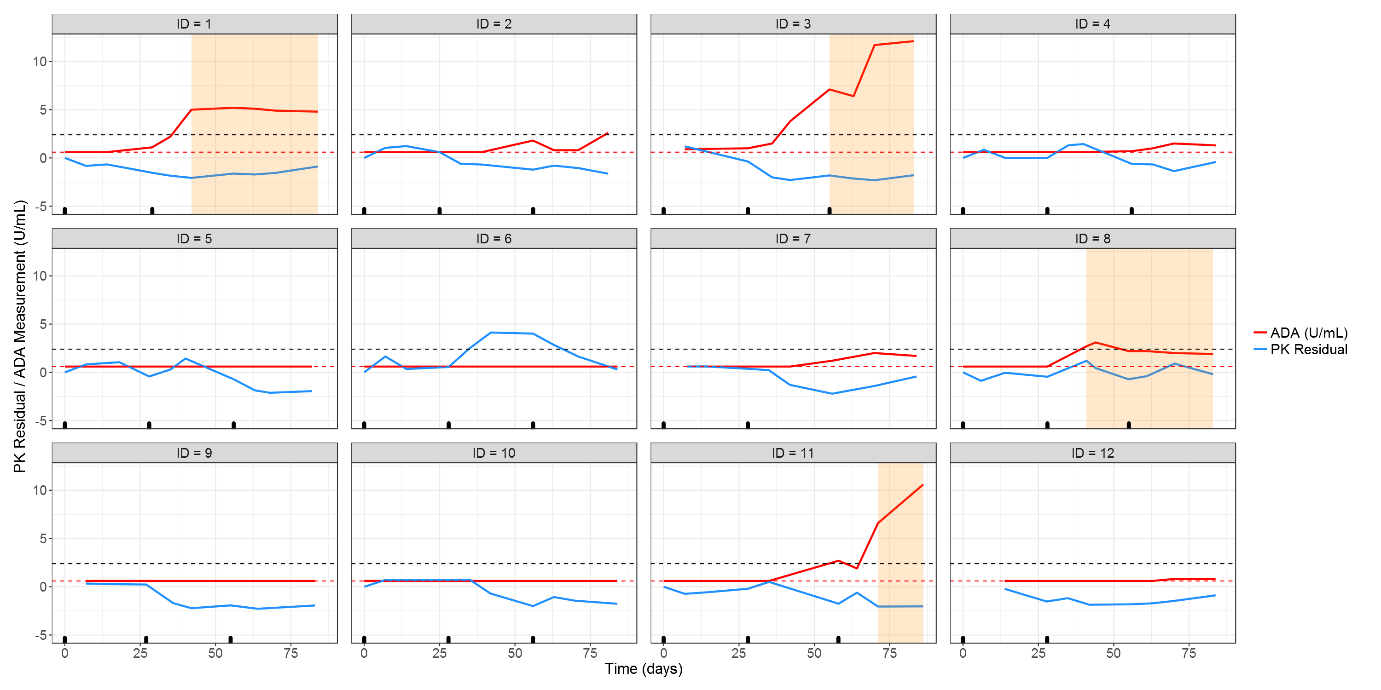
**

***Figure S3-2:*** *Individual state predictions in ten individuals resulting from model 2. Individual weighted residuals (PK residual, blue) and ADA measurements (red) are presented for 12 selected individuals. Each individual is represented in each panel. The black dashed line indicates the threshold for clinical positivity (2.4 IU/mL) for the ADA measurement and the red dashed line is the lower limit of quantification for ADA (0.6 IU/mL). The black tick marks are dosing events. The orange shaded area indicates time points which the models predicted a state associated with the production of ADA (S_ADA_).*

**Model 3 individual state sequence predictions (with *PK_RES_*)**

**
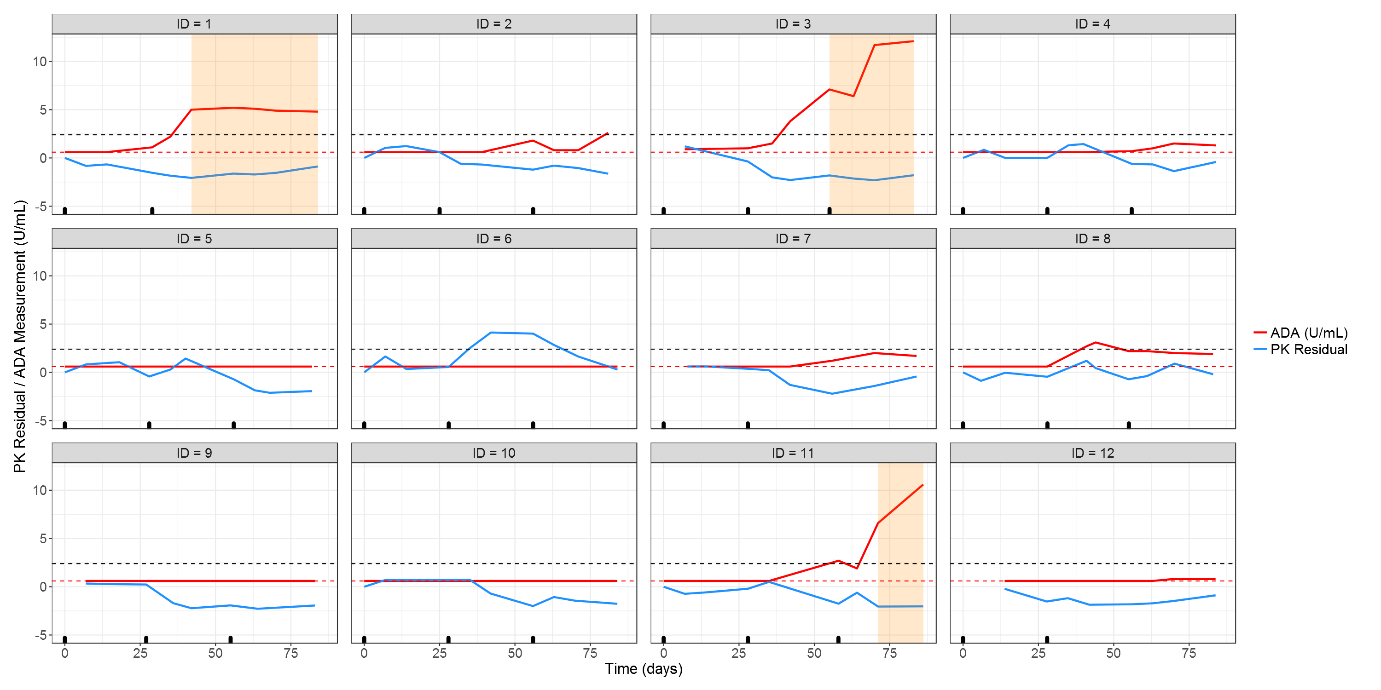
**

*Figure S3-3: Individual state predictions in ten individuals resulting from model 3. Individual weighted residuals (PK residual, blue) and ADA measurements (red) are presented for 12 selected individuals. Each individual is represented in each panel. The black dashed line indicates the threshold for clinical positivity (2.4 IU/mL) for the ADA measurement and the red dashed line is the lower limit of quantification for ADA (0.6 IU/mL). The black tick marks are dosing events. The orange shaded area indicates time points which the models predicted a state associated with the production of ADA (S_ADA_).*

**Model 4 individual state sequence predictions (with *PK_RES_*)**

**
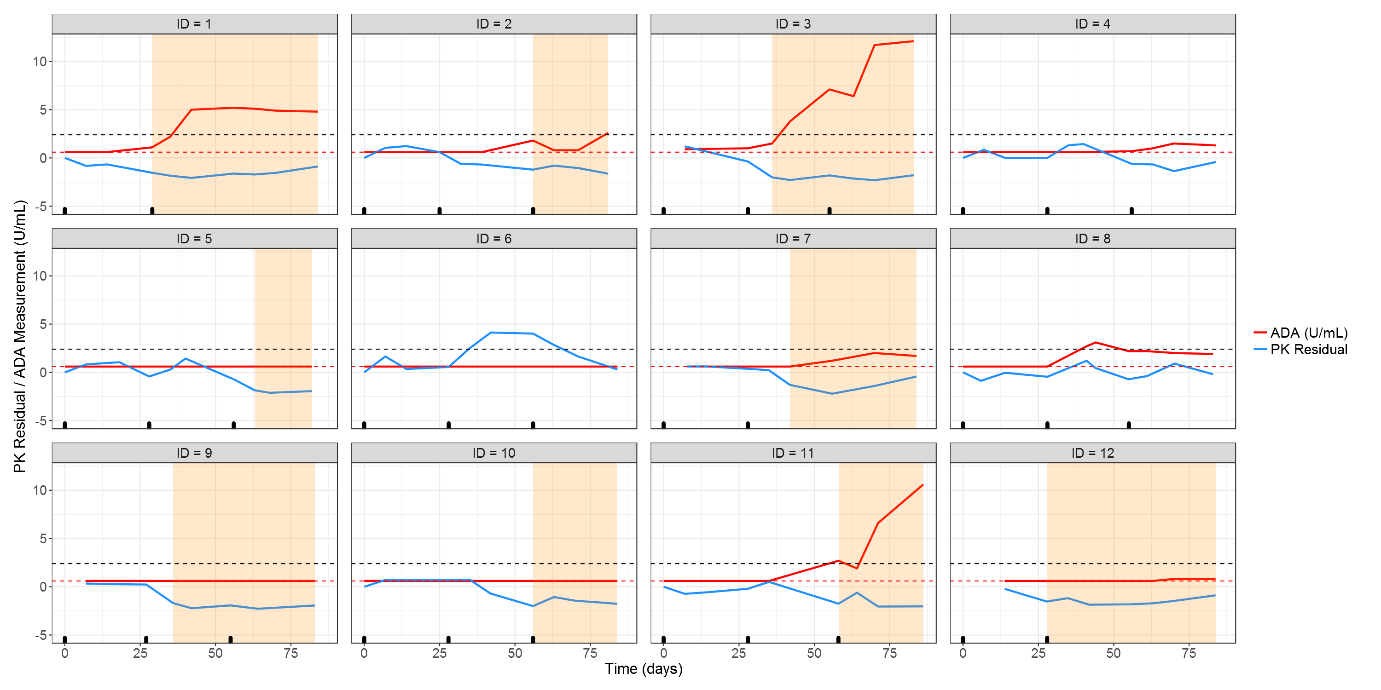
**

***Figure S3-4:*** *Individual state predictions in ten individuals resulting from model 4. Individual weighted residuals (PK residual, blue) and ADA measurements (red)) are presented for 12 selected individuals. Each individual is represented in each panel. The black dashed line indicates the threshold for clinical positivity (2.4 IU/mL) for the ADA measurement and the red dashed line is the lower limit of quantification for ADA (0.6 IU/mL). The black tick marks are dosing events. The orange shaded area indicates time points which the models predicted a state associated with the production of ADA (S_ADA_).*

**Model 5 individual state sequence predictions (with *PK_RES_*)**

**
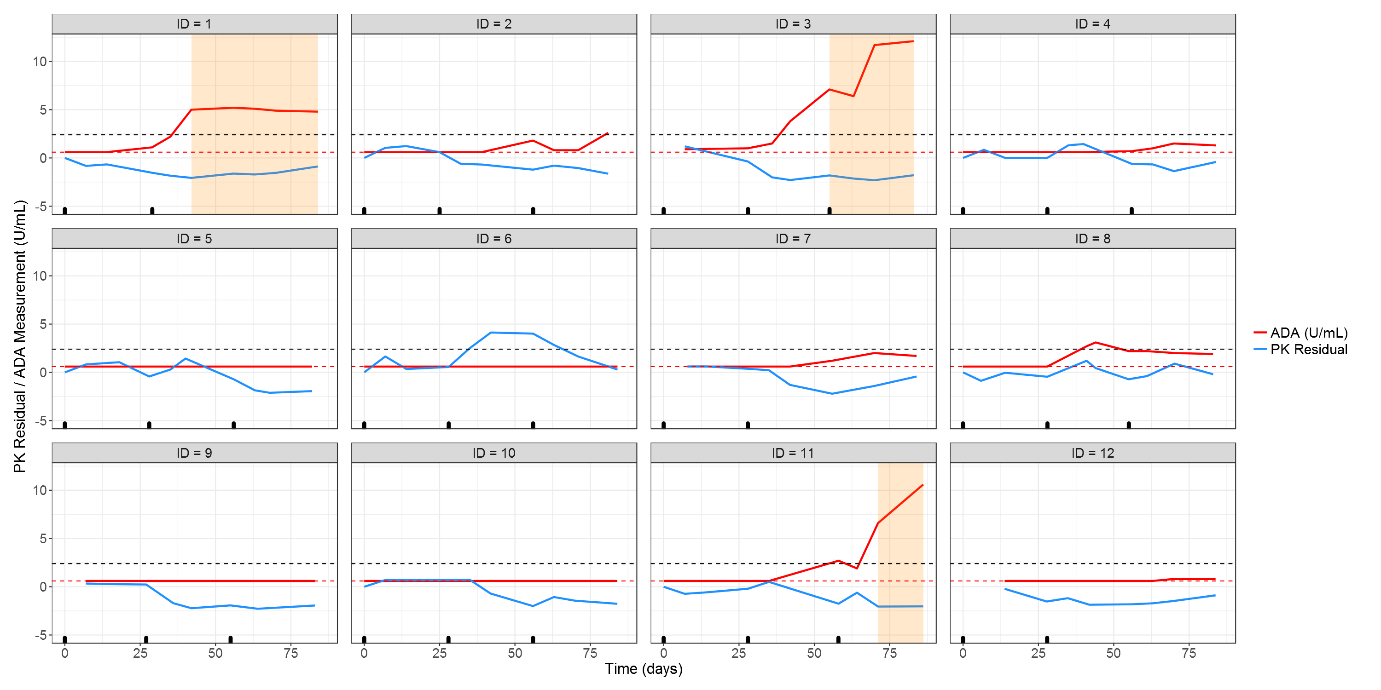
**

***Figure S3-5:*** *Individual state predictions in ten individuals resulting from model 5. Individual weighted residuals (PK residual, blue) and ADA measurements (red) are presented for 12 selected individuals. Each individual is represented in each panel. The black dashed line indicates the threshold for clinical positivity (2.4 IU/mL) for the ADA measurement and the red dashed line is the lower limit of quantification for ADA (0.6 IU/mL). The black tick marks are dosing events. The orange shaded area indicates time points which the models predicted a state associated with the production of ADA (S_ADA_).*

**Model 6 individual state sequence predictions (with *PK_RES_*)**

**
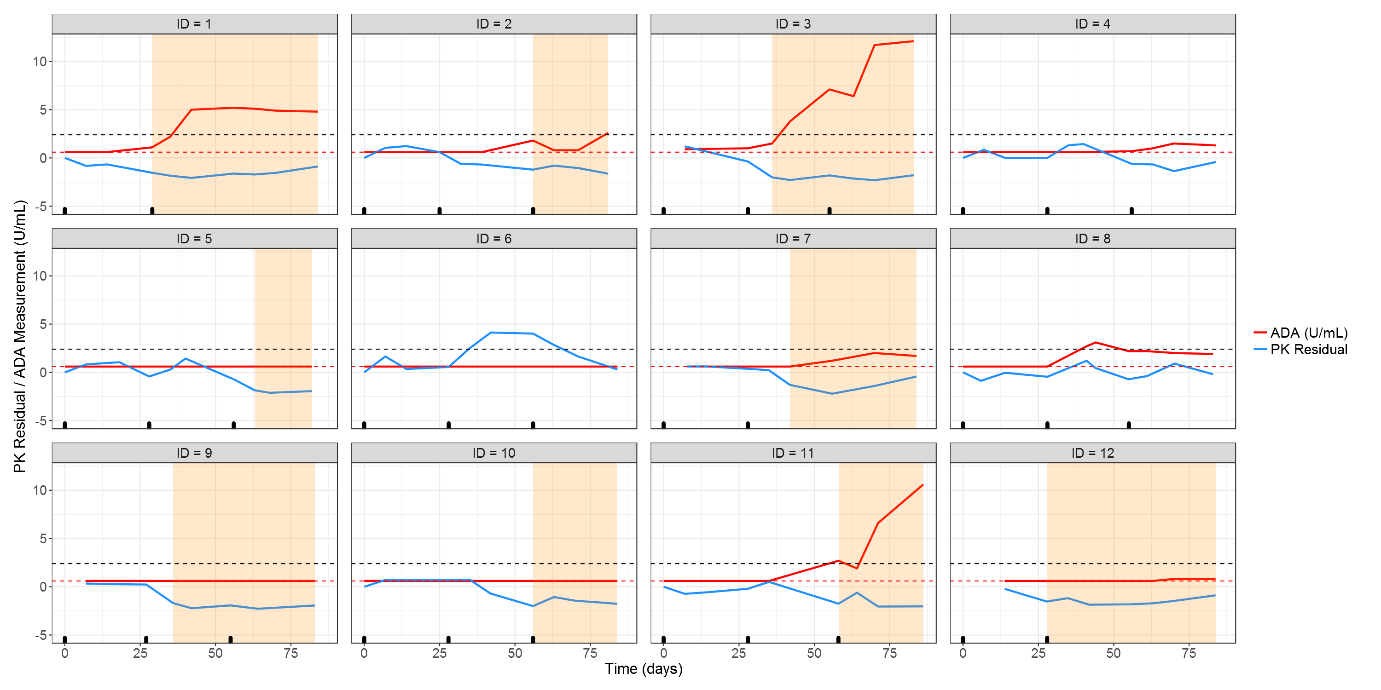
**

***Figure S3-6:*** *Individual state predictions in ten individuals resulting from model 6. Individual weighted residuals (PK residual, blue) and ADA measurements (red) are presented for 12 selected individuals. Each individual is represented in each panel. The black dashed line indicates the threshold for clinical positivity (2.4 IU/mL) for the ADA measurement and the red dashed line is the lower limit of quantification for ADA (0.6 IU/mL). The black tick marks are dosing events. The orange shaded area indicates time points which the models predicted a state associated with the production of ADA (S_ADA_).*

**Supplementary information 4**


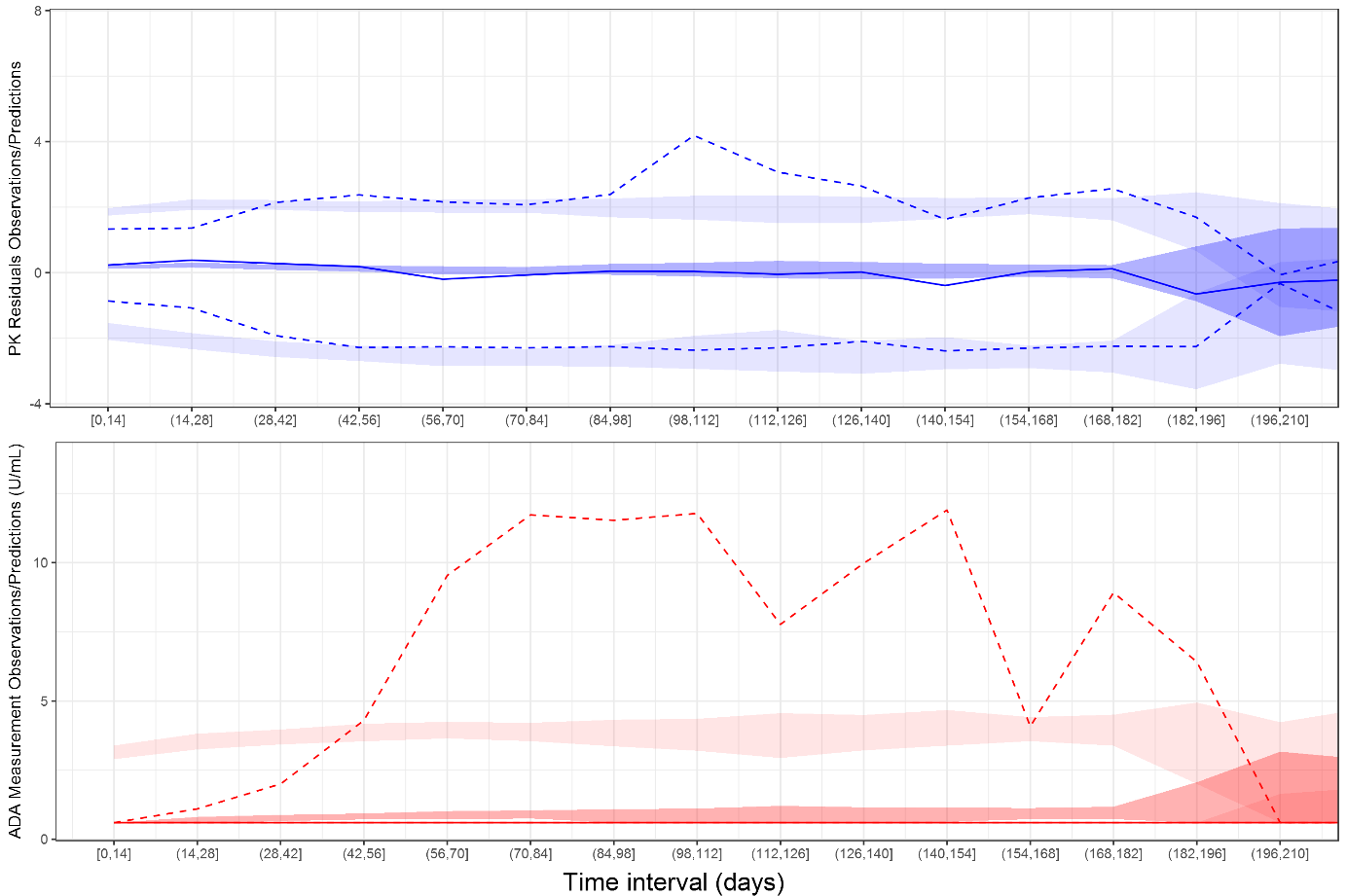


***Figure S4-1:*** *Visual predictive check illustrating predictive performance of model 6. Individual weighted residuals (PK residual, blue, top) and ADA measurements (red, bottom) are presented. Solid and dotted lines represent observed medians and 2.5^th^ and 97.5^th^ percentiles, respectively. The dark shaded area is the 95% confidence interval of the simulated median and the lighter shaded areas are the 95% confidence interval of the simulated percentiles.*


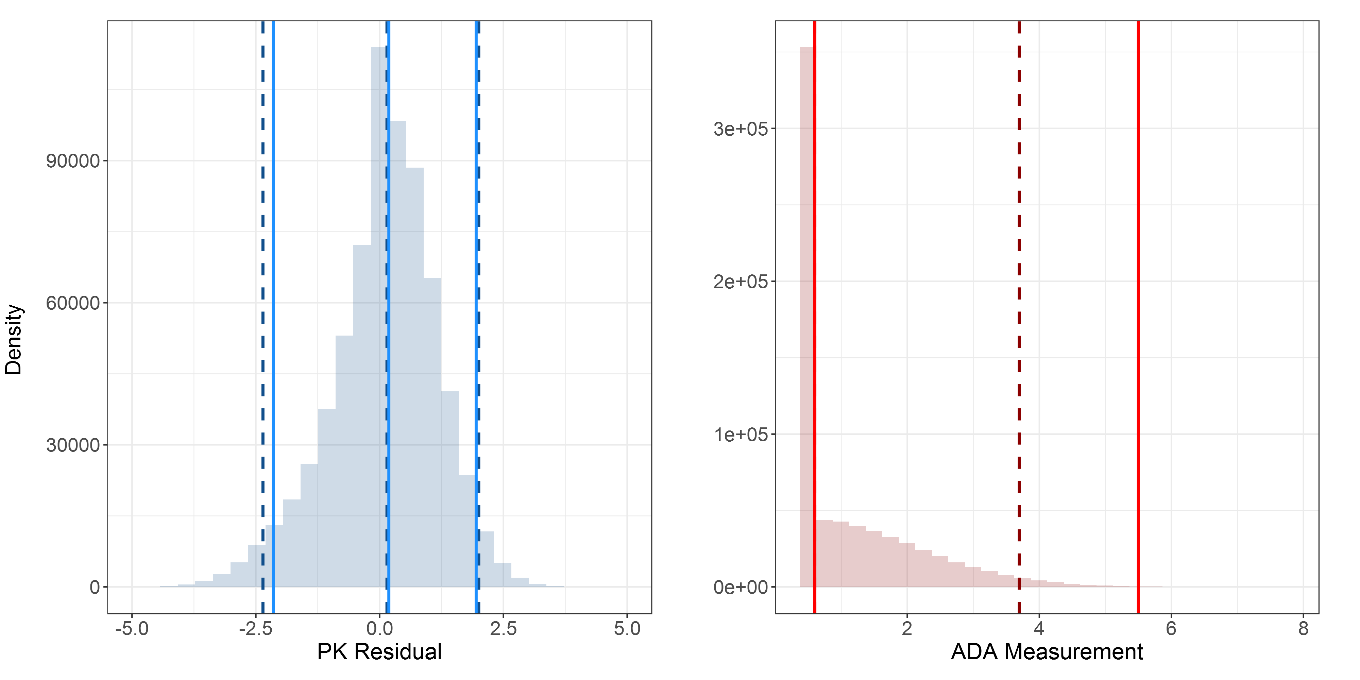


***Figure S4-2:*** *Histograms showing the simulated variables (PK_RES_, left panel and ADA measurement, right panel) from model 6. The solid lines are the observed median, 97.5^th^ and 2.5^th^ quantiles of the observed data. Note that for the observed and simulated ADA measurements the 2.5^th^ quantiles and medians are the same value (0.6). The dashed lines are the medians, 97.5^th^ and 2.5^th^ quantiles of the simulated data.*  *Simulated ADA measurements below 0.6 were set to 0.6 as LOQ.*
